# Haemosiderin-Laden Sputum Macrophages for Diagnosis in Pulmonary Veno-Occlusive Disease

**DOI:** 10.1371/journal.pone.0115219

**Published:** 2014-12-12

**Authors:** Heidi Lederer, Bettina Muggli, Rudolf Speich, Ula Treder, Hans Stricker, Jeroen Goede, Silvia Ulrich, Simon F. Stämpfli, Alexander Breitenstein

**Affiliations:** 1 Zurich Pulmonary Hypertension Program, Clinic for Internal Medicine, University Hospital Zurich, Zurich, Switzerland; 2 Cardiology, University Heart Center, University Hospital Zurich, Zurich, Switzerland; 3 Ospedale La Carità, Via all' Ospedale, Locarno, Switzerland; 4 Department of Hematolgy, University Hospital Zurich, Zurich, Switzerland; 5 Department of Cardiology, St Bartholomew's Hospital, Barts Health NHS Trust, London, United Kingdom; Vanderbilt University Medical Center, United States of America

## Abstract

**Aims:**

Pulmonary veno-occlusive disease (PVOD) is a rare condition of pulmonary arterial hypertension (PAH), in which post-capillary veins are affected. Since the therapeutic approach in PVOD differs from other forms of PAH, it is crucial to establish the diagnosis. Due to the fact that affected patients are often hemodynamically unstable, minimal invasive procedures are necessary for the diagnostic work-up. Chronic alveolar haemorrhage has been observed during bronchoalveolar lavage in PVOD cases. This study therefore investigates whether signs of alveolar haemorrhage can also be found in the sputum of these patients.

**Methods and Results:**

Six patients suffering from PVOD were included in this analysis. As controls, patients with idiopathic PAH (n = 11), chronic thromboembolic PH (n = 9) and with sclerodermia-associated PH (n = 10) were assessed. Sputum from every patient was obtained by a non-invasive manner. The amount of haemosiderin-laden macrophages was determined using the Golde score. There were statistically significant more haemosiderin-laden macrophages in the sputum of patients suffering from PVOD as compared to the other groups (*P*<0.05). Assuming a cut-off of 200 on the Golde score, all of the 6 PVOD patients surpassed this value compared with only 1 out of the 30 cases with precapillary PH. Thus, sensitivity and specificity with respect to the diagnosis of PVOD was 100% and 97%, respectively.

**Conclusion:**

The content of haemosiderin-laden macrophages in the sputum of patients suffering from PVOD is significantly higher as compared to other forms of PH and may be useful in the non-invasive diagnostic work-up of these patients.

## Introduction

Pulmonary hypertension (PH) is a severe condition characterized by elevated mean pulmonary arterial pressure ≥25 mmHg resulting in right heart failure and death [1]. According to recent guidelines, PH can be classified based on clinical and haemodynamic criteria. One distinct subgroup of pulmonary arterial hypertension (PAH) is pulmonary veno-occlusive disease (PVOD) [2], [3], which accounts for only 5–10% of all cases of PAH [4]. In contrast to the involvement of small pre-capillary pulmonary arteries as in PAH, vascular pathology in PVOD is characterized by fibrous remodeling of post-capillary septal veins and praeseptal venules [5], [6].

Clinical presentation of PVOD is non-specific and very similar to PAH. Hence, distinguishing PVOD from PAH based on clinical criteria is often difficult. Nevertheless, since PVOD has a worse prognosis than the various forms of PAH and may exhibit a poor and even adverse response to common vasodilator therapies which may lead to severe and sometimes fatal pulmonary oedema, it is of crucial importance to identify cases of PVOD. In addition, a diagnosis of PVOD has serious therapeutic consequences since such patient qualify for lung transplantation and should be listed as soon as possible.

PVOD can be suspected by the constellation of significant hypoxaemia, a very low carbon monoxide diffusion capacity (DLCO) as well as characteristic radiological features on chest computed tomography [7]–[9]. The definitive diagnosis of PVOD would require surgical lung biopsy, but such an invasive approach is not feasible in these high-risk patients. Hence, less invasive diagnostic tools would be helpful. As mentioned, PVOD affects the post-capillary veins and may hence result in engorgement of the capillaries leading to occult alveolar haemorrhage. Indeed, a study of bronchoalveolar lavage (BAL) in patients suffering from PVOD could demonstrate a significantly higher percentage of haemosiderin-laden macrophages as compared to other form of PH [10]. Hence, BAL has become an additional investigation tool to diagnose PVOD. Nevertheless, even though BAL has been reported to be a safe intervention in stable PH patients [10], it is an invasive procedure and may carry an increased risk of respiratory impairment and hemodynamic instability. Thus, there is an urgent need for a less invasive procedure. The aim of the current study is to investigate whether the analysis of haemosiderin-laden macrophages in the sputum of patients, might represent an additional diagnostic tool in the work-up for PVOD comparable to BAL.

## Materials and Methods

### Patient data

The study was conducted at the Pulmonary Hypertension Unit, University Hospital Zurich, Switzerland, between July 2003 and November 2011. PVOD was assumed with a mean pulmonary arterial pressure of ≥25 mmHg, a pulmonary arterial wedge pressure ≤15 mmHg and the presence of at least 2 out of 3 chest computed tomography findings (centrilobular ground-glass opacities, septal lines and mediastinal lymph node enlargement) [11], as well as laboratory findings suggestive for PVOD, i.e. a reduced diffusion capacity of the lungs for carbon monoxide (DLCO; <50%) and hypoxaemia at rest with a PaO_2_<8 kPa, and clinical sings like crackles on auscultation or pulmonary oedema after vasodilator therapy [8], [9], [12]. Pathological confirmation of PVOD could be obtained in only one patient from the explanted lungs after lung transplantation.

As control groups, patients suffering from idiopathic PAH (IPAH; n = 11) [13], [14], chronic thromboembolic PH (CTEPH; n = 9) [13]–[15], and scleroderma-associated PAH (SPAH; n = 10) [13], [14], [16] were included in this analysis. The diagnosis of these entities was established according to current guidelines [13]–[16].

Ethical approval was granted by the institutional ethical committee (“Kantonale Ethikkommission Zürich”). All subjects signed an informed consent form.

### Sputum collection and processing

**Figure 1:**
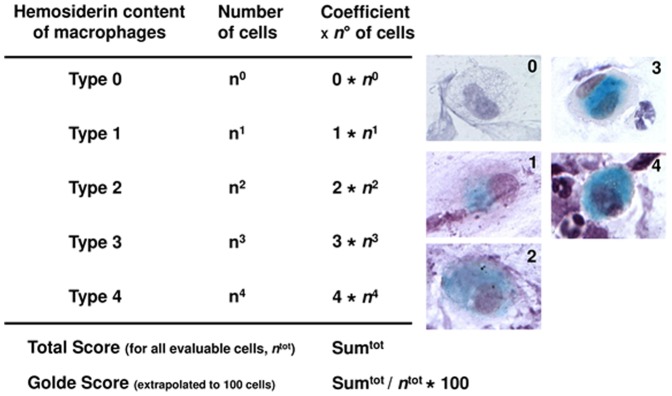
Description of the Golde score calculation in sputum macrophages.

Sputum from every patient was obtained by a non-invasive manner without any induction by hypertonic saline solution. Per visit, each patient has given three probes of sputum which where stained for haemosiderin by the Prussian blue procedure. The patients were encouraged as far as possible to produce a viable sputum sample. However, after 5 attempts the procedure was halted in consideration of the reduced general condition of these patients. The slides were stored and then examined by two physicians unaware of the clinical course of the patients (H.L., B.M.). Quality criteria for sputum was presence of more than 25 macrophages on the slides. The haemosiderin content of alveolar macrophages was estimated according to the Golde scoring system [17]. Each macrophage was scored for haemosiderin content using the following scale: 0, no color; 1, faint blue in a part or the whole cytoplasm without or with single deep blue granules; 2, medium color intensity throughout the cytoplasm without or with deep blue granules in minor (<50%) portions of the cytoplasm; 3, deep blue granules in major (>50%) portions of the cytoplasm; and 4, deep blue granules throughout the cell (Fig. 1). The total score was extrapolated to an average of 100 cells.

### Statistical analysis

Continuous variables are expressed as median (interquartile range) and frequencies for categorical variables. The Golde score was analyzed by the Kruskal-Wallis One-Way analysis of variance, the Spearman's coefficient of correlation was used to assess inter-observer variation in the analysis of the Golde score. Clinical data was analyzed by the χ^2^ test for categorical and by the Kruskal-Wallis One-Way analysis for continuous data. The level of significance was defined as a two-tailed *P* value <0.05. All statistical analyses were performed with SPSS Statistics 19.0 for Windows (SPSS, Inc. 2010).

## Results

### Patient's characteristics

36 subjects were included in the present study: IPAH (n = 11), CTEPH (n = 9), SAPH (n = 10), and PVOD (n = 6). A detailed overview of the patient's characteristics is summarized in table 1. Briefly, there was a non-significant trend towards higher age in the PVODs (70±6 years in PVOD versus 55±20 years for IPAH, 63±16 years for CTEPH, and 67±10 years for SAPH, respectively; *P* = NS), while there was a female predominance in the SAPH group as compared to the others (100% female in SAPH versus 36.4% for IPAH, 44.4% for CTEPH, and 20.0% for PVOD; *P*<0.05). Dyspnea was the main symptom in all patients with the majority suffering from New York Heart association functional class III-IV. The 6-minute walking distance (6-MWD) as well as the mean pulmonary artery pressure and the cardiac index did not differ significantly between the groups.

**Table 1:** Clinical characteristics of the four patient groups.

	Group			
	IPAH (n = 11)	CTEPH (n = 9)	SAPH (n = 10)	PVOD (n = 6)
**Gender [m/f]**	7/4	5/4	0/10	5/1
**Age [years]**	55±20	63±16	67±10	70±6
**Smoking [%]**	36	13	10	33
**NYHA functional class [n]**				
**I-II**	2/11	4/9	2/10	1/6
**III-IV**	9/11	5/9	8/10	5/6
**Baseline 6-MWD [m]**	292±127	431±183	359±160	295±74
**Heart rate [bpm]**	77±10	92±17	78±16	82±13
**Systolic blood pressure [mmHg]**	117±17	134±24	118±15	119±20
**Diastolic blood pressure [mmHg]**	68±8	74±14	68±12	71±11
**Mean pulmonary artery pressure [mmHg]**	44±16	45±17	32±12	39±11
**Pulmonary artery wedge pressure [mmHg]**	13±10	11±7	13±5	8±3
**Central venous pressure [mmHg]**	9±5	8±6	7±5	6±4
**Cardiac index [l/min/m^2^]**	3.6±1.3	2.6±0.5	3.1±1.1	2.8±0.5
**Mixed venous oxygen saturation [%]**	65.2±9.1	62.7±9.7	73.0±6.7	60.4±14.2
**Pulmonary vascular resistance [WU]**	6.2±3.1	9.6±5.6	4.6±2.9	6.8±3.5
**Systemic vascular resistance [WU]**	16.0±3–6	21.7±5.9	19.2±6.0	15.2±1.7

In table 2, the clinical features of the 6 patients with suspected PVOD are shown. All of them demonstrated at least 4 findings characteristically associated with PVOD from data of the literature, including at least 2 of the radiologic criteria.

**Table 2:** Clinical features suggesting the presence of PVOD, number of evaluable cells, and Golde score.

Patient #	Sex	Age [years]	NYHA functional class	6-MWD [m]	mPAP [mmHg]	CI [l/min/m^2^]	PVR [WU]	PAWP [mmHg]	DLCO [%]	PaO_2_ [kPa]	Lymphadenopathy	Ground-glass opacity	Septal lines	Crackles or pulmonary oedema	Number of PVOD features	Number of sputum cells	Golde score
1	M	74	III	296	32	2.9	4.3	11	47	6.78	+	+	+	-	5	48	264
2	M	74	III	372	31	3.0	3.8	9	23	5.32	+	-	+	+	5	143	270
3	F	65	II	158	38	2.7	7.6	4	47	6.62	+	+	-	-	4	169	263
4	M	74	III	338	32	2.4	5.0	9	39	6.25	+	-	+	-	4	315	240
5	M	60	III	322	38	3.6	6.8	5	33	6.83	+	-	+	-	4	250	315
6	M	71	III	282	60	2.2	13.3	10	48	7.74	+	+	+	+	6	212	252

6-MWD: 6-minute walking distance. mPAP: mean pulmonary artery pressure. CI: cardiac index. PVR: pulmonary vascular resistance. WU: Wood units. PAWP: pulmonary artery wedge pressure. DLCO: diffusion capacity. PaO_2_: partial pressure of oxygen in arterial blood.

### Amount of haemosiderin-laden macrophages is higher in PVOD as compared to the other forms of precapillary PH

**Figure 2:**
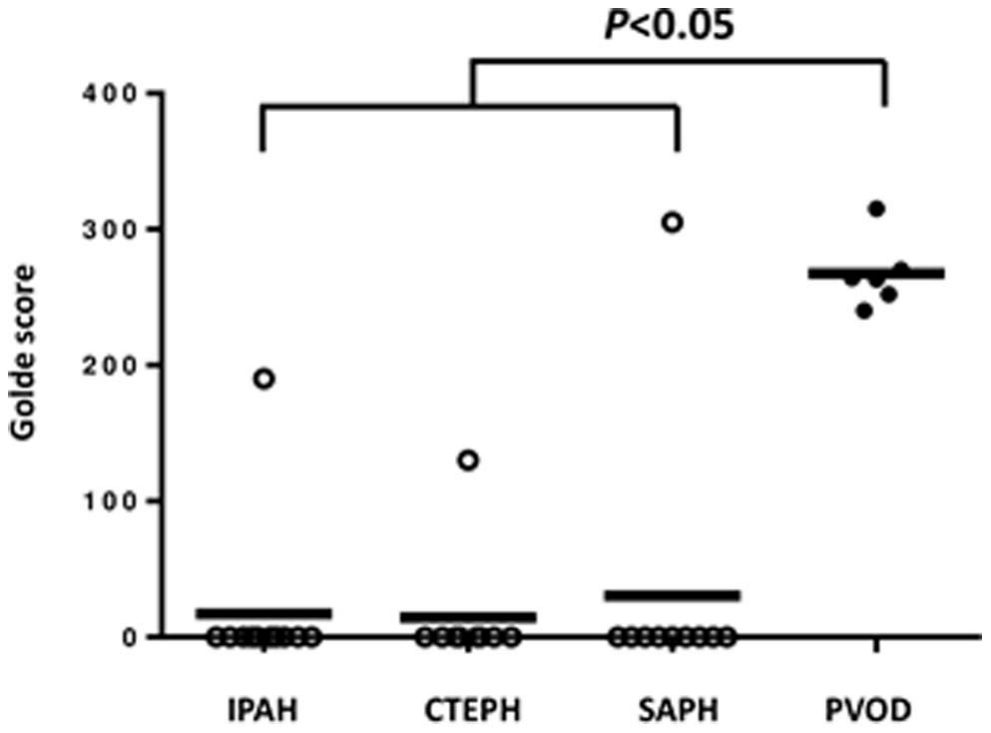
Haemosiderin-laden macrophages are significantly (*P*<0.05) more present in sputum from PVOD patients as compared to the others (IPAH, CTEPH and SAPH, respectively).

All slides were examined for haemosiderin-laden macrophages by two blinded physicians unaware of the clinical data. The method to calculate the Golde score is summarized in Fig. 1. The mean of the evaluable sputum macrophages was 267 (median 263; range 240–315). An interobserver variation between the two investigators with respect to the number of identifiable sputum cells as well as to the Golde score could be excluded according to the Spearman's coefficient of correlation (r>0.9). There was no difference in the content of haemosiderin-laden macrophages between IPAH, CTEPH and SPAH (*P* = NS; Fig. 2). However, there were statistically significant more haemosiderin-laden macrophages in the sputum of patients suffering from PVOD demonstrated by a Golde score>200 as compared to the other groups (*P*<0.05; Fig. 2). All PVOD patients with evaluable cells in their sputum had a Golde score >200. Assuming a cut-off value for the Golde score of 200, the sensitivity for the diagnosis of clinical PVOD was 100% and the specificity 97%, respectively.

**Figure 3:**
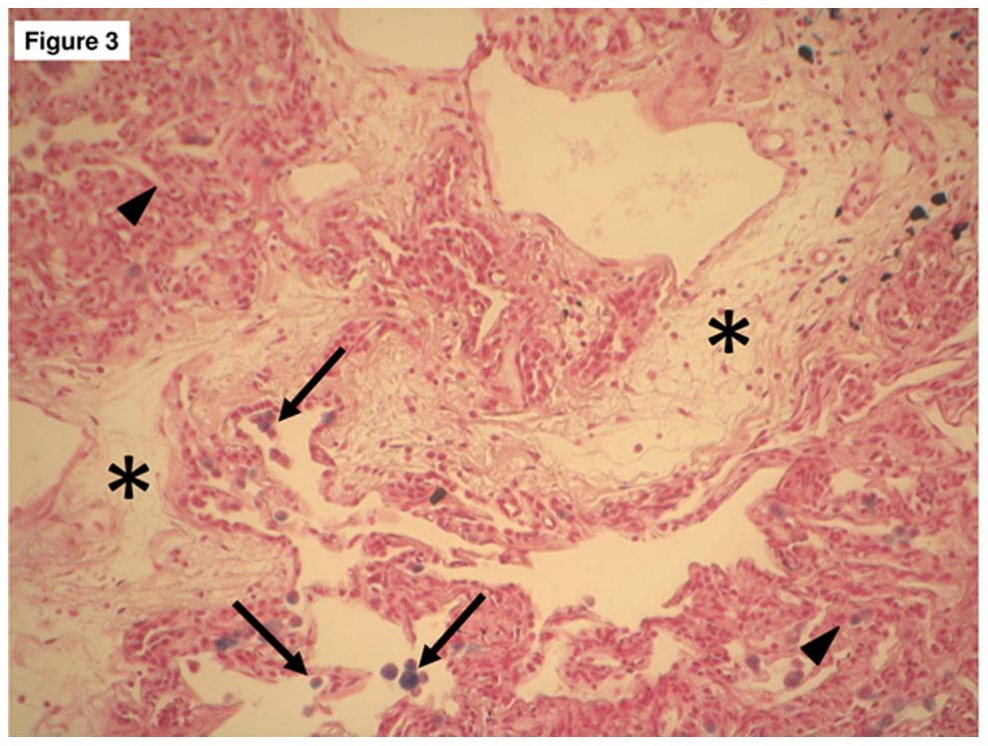
Histologic specimen from a patient suffering from PVOD who underwent lung transplantation. Intimal thickening and fibrous obstruction of septal veins and preseptal venules (asterixis) as well as features of pulmonary capillary hemangiomatosis with proliferation of dilated and congested capillaries as well as dilated lymphatic (left upper and right lower part of the figure; arrowhead). Alveoli with haemosiderin-laden macrophages of Golde score 3–4 (arrow) are clearly visible. Haemosiderin-negativ macrophages are difficult to be identified. Golde score from the tissue was 304, as compared to 252 in the sputum.

For comparison with BAL, we could retrieve only one sample from patient 1 (which was obtained one year before the sputum). The Golde score at that time point was 312, i.e. comparable to the count of 264 found in the sputum. Since all patients except one did not qualify for lung transplantation, we could only assess one histological specimen. Interestingly, this patient also showed features of capillary hemangiomatosis (Fig. 3). The Golde score calculated from the tissue was 304 and hence comparable to the count of 252 found in the sputum.

## Discussion

The present analysis demonstrates for the first time that the content of haemosiderin-laden macrophages in the sputum is significantly higher in patients suffering from PVOD as compared to other forms of PH. The sensitivity and specificity for the diagnosis of clinical PVOD was 100% and 97%, respectively. Hence, sputum examination for haemosiderin-laden macrophages may be considered as an additional non-invasive diagnostic piece of puzzle in the work-up of this serious disease. In addition, our study confirms the previous finding of Rabiller and colleagues, who could demonstrate comparable findings by examining alveolar macrophages from BAL [10].

PVOD is a rare cause of PAH accounting for less than 5–10% of all PAH cases [18]. According to the recent guidelines, PVOD is considered as a subgroup of PAH [3]. On one side, the clinical presentation resembles that of PAH including the presence of similar risk factors such as HIV infection. In contrast, the main pathophysiological difference is the fibrous remodeling of post-capillary septal veins and praeseptal venules which is not present in other forms of PAH except to some extent in SPAH [19], [20]. Importantly, the involvement of post-capillary vessels allows a histological differentiation between the different entities of PH and also has important implications regarding the therapeutic options. Particularly the use of intravenous as well as oral vasodilators, which are commonly used in the treatment of PAH, may be dangerous due to the risk for development of pulmonary oedema in these patients suffering from PVOD [8], [20]–[23]. Therefore, to establish the correct diagnosis is crucial to offer optimal treatment for these patients.

Since lung biopsy is usually not possible in PVOD subjects because it is considered a high-risk procedure in severe PH, one has to rely on clinical features and non-invasive examinations outlined in the algorithm for PVOD management of the French Reference Center for Pulmonary Hypertension[8]. In this respect, sputum analysis for haemosiderin-laden macrophages might represent an asset in the diagnostic puzzle. Using a crude Bayesian approach without consideration of dependency of the variables, the likelihood ratios (LHR) of the single parameters can be calculated. For instance, according to the data of Montani and colleagues, the presence of septal lines in a chest computed tomography has a positive (LHR+) and a negative LHR (LHR-) of 4.3 and 0.41, respectively. Due to our data, the LHR+ and LHR- with respect to the presence of a Golde score >200 are 30.00 and 0.00, respectively. Hence, if both parameters would turn to be positive, by multiplying the ratios, the post-test detection rate for a PVOD would be around 129∶1. Hence, this study clearly shows that collection of sputum for the analysis of haemosiderin-laden macrophages could be used as an important tool in the diagnostic work-up of PVOD.

Of course, the current case series has its limitations. First, due to the fact that PVOD is a very rare variant of an orphan disease with a prevalence of only 15 patients per one million habitants [18], the number of included patients is low (n = 6). Nevertheless, this has to been put into the context that up to the year 2006, only about 200 cases of PVOD have been reported in the literature. Secondly, a histological confirmation was not obtained from all PVOD patients because lung biopsy is not recommended in these patients [20]. Notwithstanding, one patient of the current series underwent lung transplantation, and the Golde score in the tissue was comparable to that found in the sputum. In an other cased, the Golde score from the sputum was comparable to that of the BAL. Thirdly, even due to the fact that sensitivity of the sputum examination for haemosiderin-laden macrophages was statistically 100%, we would like to point out that the diagnostic work-up can further be increased by a proper and repeated sputum collection.

In summary, the present study on patients suffering from PVOD indicates that the collection of non-induced sputum for the analysis of haemosiderin-laden macrophages could be used as a non-invasive diagnostic tool for the work-up of PVOD.

## References

[1] Rubin LJ (1997) Primary pulmonary hypertension. N Engl J Med 336:111–117. 8988890 10.1056/NEJM199701093360207

[2] Simonneau G, Robbins IM, Beghetti M, Channick RN, Delcroix M, et al (2009) Updated Clinical Classification of Pulmonary Hypertension. Journal of the American College of Cardiology 54:S43–S54. 19555858 10.1016/j.jacc.2009.04.012

[3] Galie N, Hoeper MM, Humbert M, Torbicki A, Vachiery JL, et al (2009) Guidelines for the diagnosis and treatment of pulmonary hypertension: the Task Force for the Diagnosis and Treatment of Pulmonary Hypertension of the European Society of Cardiology (ESC) and the European Respiratory Society (ERS), endorsed by the International Society of Heart and Lung Transplantation (ISHLT). Eur Heart J 30:2493–2537. 19713419 10.1093/eurheartj/ehp297

[4] Mandel J, Mark EJ, Hales CA (2000) Pulmonary veno-occlusive disease. Am J Respir Crit Care Med 162:1964–1973. 11069841 10.1164/ajrccm.162.5.9912045

[5] Huertas A, Girerd B, Dorfmuller P, O'Callaghan D, Humbert M, et al**.** (2011) Pulmonary veno-occlusive disease: advances in clinical management and treatments. Expert Rev Respir Med 5: 217–229; quiz 230–211. 10.1586/ers.11.15 21510732

[6] Pietra GG, Capron F, Stewart S, Leone O, Humbert M, et al (2004) Pathologic assessment of vasculopathies in pulmonary hypertension. Journal of the American College of Cardiology 43:S25–S32. 10.1016/j.jacc.2004.02.033 15194175

[7] Palmer SM, Robinson LJ, Wang A, Gossage JR, Bashore T, et al (1998) Massive pulmonary edema and death after prostacyclin infusion in a patient with pulmonary veno-occlusive disease. Chest 113:237–240. 9440597 10.1378/chest.113.1.237

[8] Montani D, Achouh L, Dorfmuller P, Le Pavec J, Sztrymf B, et al (2008) Pulmonary veno-occlusive disease: clinical, functional, radiologic, and hemodynamic characteristics and outcome of 24 cases confirmed by histology. Medicine (Baltimore) 87:220–233. 18626305 10.1097/MD.0b013e31818193bb

[9] Holcomb BW Jr, Loyd JE, Ely EW, Johnson J, Robbins IM (2000) Pulmonary veno-occlusive disease: a case series and new observations. Chest 118:1671–1679. 11115457

[10] Rabiller A, Jais X, Hamid A, Resten A, Parent F, et al (2006) Occult alveolar haemorrhage in pulmonary veno-occlusive disease. Eur Respir J 27:108–113. 16387942 10.1183/09031936.06.00054105

[11] Resten A, Maitre S, Humbert M, Rabiller A, Sitbon O, et al (2004) Pulmonary hypertension: CT of the chest in pulmonary venoocclusive disease. AJR Am J Roentgenol 183:65–70. 15208112 10.2214/ajr.183.1.1830065

[12] Elliott CG, Colby TV, Hill T, Crapo RO (1988) Pulmonary veno-occlusive disease associated with severe reduction of single-breath carbon monoxide diffusing capacity. Respiration 53:262–266. 3175350 10.1159/000195438

[13] Zamanian RT, Kudelko KT, Sung YK, de Jesus Perez V, Liu J, et al (2014) Current clinical management of pulmonary arterial hypertension. Circ Res 115:131–147. 24951763 10.1161/CIRCRESAHA.115.303827 PMC4452016

[14] Simonneau G, Gatzoulis MA, Adatia I, Celermajer D, Denton C, et al (2013) Updated clinical classification of pulmonary hypertension. J Am Coll Cardiol 62:D34–41. 24355639 10.1016/j.jacc.2013.10.029

[15] Lang IM, Madani M (2014) Update on chronic thromboembolic pulmonary hypertension. Circulation 130:508–518. 25092279 10.1161/CIRCULATIONAHA.114.009309

[16] Chaisson NF, Hassoun PM (2013) Systemic sclerosis-associated pulmonary arterial hypertension. Chest 144:1346–1356. 24081346 10.1378/chest.12-2396 PMC3787920

[17] Golde DW, Drew WL, Klein HZ, Finley TN, Cline MJ (1975) Occult pulmonary haemorrhage in leukaemia. Br Med J 2:166–168. 1125726 10.1136/bmj.2.5964.166 PMC1675967

[18] Humbert M, Sitbon O, Chaouat A, Bertocchi M, Habib G, et al (2006) Pulmonary arterial hypertension in France: results from a national registry. Am J Respir Crit Care Med 173:1023–1030. 16456139 10.1164/rccm.200510-1668OC

[19] Lantuejoul S, Sheppard MN, Corrin B, Burke MM, Nicholson AG (2006) Pulmonary veno-occlusive disease and pulmonary capillary hemangiomatosis: a clinicopathologic study of 35 cases. Am J Surg Pathol 30:850–857. 16819327 10.1097/01.pas.0000209834.69972.e5

[20] Montani D, Price LC, Dorfmuller P, Achouh L, Jais X, et al (2009) Pulmonary veno-occlusive disease. Eur Respir J 33:189–200. 19118230 10.1183/09031936.00090608

[21] Creagh-Brown BC, Nicholson AG, Showkathali R, Gibbs JS, Howard LS (2008) Pulmonary veno-occlusive disease presenting with recurrent pulmonary oedema and the use of nitric oxide to predict response to sildenafil. Thorax 63:933–934. 18820120 10.1136/thx.2007.088831

[22] Barboza CE, Jardim CV, Hovnanian AL, Dias BA, Souza R (2008) Pulmonary veno-occlusive disease: diagnostic and therapeutic alternatives. J Bras Pneumol 34:749–752. 18982212 10.1590/s1806-37132008000900015

[23] Shackelford GD, Sacks EJ, Mullins JD, McAlister WH (1977) Pulmonary venoocclusive disease: case report and review of the literature. AJR Am J Roentgenol 128:643–648. 403796 10.2214/ajr.128.4.643

